# Proteogenomic analysis of pathogenic yeast *Cryptococcus neoformans* using high resolution mass spectrometry

**DOI:** 10.1186/1559-0275-11-5

**Published:** 2014-02-03

**Authors:** Lakshmi Dhevi Nagarajha Selvan, Jyothi Embekkat Kaviyil, Raja Sekhar Nirujogi, Babylakshmi Muthusamy, Vinuth N Puttamallesh, Tejaswini Subbannayya, Nazia Syed, Aneesha Radhakrishnan, Dhanashree S Kelkar, Sartaj Ahmad, Sneha M Pinto, Praveen Kumar, Anil K Madugundu, Bipin Nair, Aditi Chatterjee, Akhilesh Pandey, Raju Ravikumar, Harsha Gowda, Thottethodi Subrahmanya Keshava Prasad

**Affiliations:** 1 Institute of Bioinformatics, International Technology Park, Bangalore 560 066, India; 2 Amrita School of Biotechnology, Amrita University, Kollam 690 525, India; 3 Department of Neuromicrobiology, National Institute of Mental Health and Neuro Sciences, Bangalore 560 029, India; 4 Centre of Excellence in Bioinformatics, School of Life Sciences, Pondicherry University, Puducherry 605 014, India; 5 Department of Biochemistry and Molecular Biology, School of Life Sciences, Pondicherry University, Puducherry 605 014, India; 6 Manipal University, Madhav Nagar, Manipal 576 104, India; 7 McKusick-Nathans Institute of Genetic Medicine, Johns Hopkins University School of Medicine, Baltimore, MD 21205, USA; 8 Department of Biological Chemistry, Johns Hopkins University School of Medicine, Baltimore, MD 21205, USA; 9 Department of Pathology, Johns Hopkins University School of Medicine, Baltimore, MD 21205, USA; 10 Department of Oncology, Johns Hopkins University School of Medicine, Baltimore, MD 21205, USA

**Keywords:** Fungal infection, Fungal genomics, Antifungal drugs, Cryptococcal meningitis, Computational prediction, Genome annotation

## Abstract

**Background:**

*Cryptococcus neoformans,* a basidiomycetous fungus of universal occurrence, is a significant opportunistic human pathogen causing meningitis. Owing to an increase in the number of immunosuppressed individuals along with emergence of drug-resistant strains, *C. neoformans* is gaining importance as a pathogen. Although, whole genome sequencing of three varieties of *C. neoformans* has been completed recently, no global proteomic studies have yet been reported.

**Results:**

We performed a comprehensive proteomic analysis of *C. neoformans* var. *grubii* (Serotype A), which is the most virulent variety, in order to provide protein-level evidence for computationally predicted gene models and to refine the existing annotations. We confirmed the protein-coding potential of 3,674 genes from a total of 6,980 predicted protein-coding genes. We also identified 4 novel genes and corrected 104 predicted gene models. In addition, our studies led to the correction of translational start site, splice junctions and reading frame used for translation in a number of proteins. Finally, we validated a subset of our novel findings by RT-PCR and sequencing.

**Conclusions:**

Proteogenomic investigation described here facilitated the validation and refinement of computationally derived gene models in the intron-rich genome of *C. neoformans*, an important fungal pathogen in humans.

## Background

*Cryptococcus neoformans* is an opportunistic human pathogen, which causes cryptococcal meningitis, mostly among immune-impaired individuals [1,2]. *Cryptococcus neoformans* species complex comprises of two subspecies (*C. neoformans* and *C. gattii*), two varieties (*C. neoformans* var. *grubii* and *C. neoformans* var. *neoformans*) and five serotypes – Serotype A (*C. neoformans* var. *grubii*), Serotype D and Serotype AD (*C. neoformans* var. *neoformans*) and Serotype B and C (*C. gattii*) [3,4]. Whole genome sequencing of *C. neoformans* var. *neoformans*[5], *C. neoformans* var. *grubii*[6] and *C. gattii*[7] has been carried out recently. *C. neoformans* var. *grubii* (Serotype A) is the predominant disease-causing variety worldwide and accounts for about 95% of cryptococcal infections [8,9]. The nuclear genome of *C. neoformans* var. *grubii* is approximately 19 Mb in size, which is organized into 14 chromosomes predicted to encode 6,967 protein-coding genes [6]. More than 98% of these protein-coding genes contain short introns [10]. These introns add to the complexity of genome through alternative splicing, exon skipping or truncation/extension at their 5′ or 3′ ends. Therefore, experimental approaches are required in order to refine these computationally derived gene models. cDNA libraries have been used to verify some of the gene models in *C. neoformans* var. *grubii*[11,12]. In this study, we used high accuracy mass spectrometry-derived data as a complementary approach to validate and improve the annotation of *C. neoformans* var. *grubii.*

Proteogenomics complements other genome annotation methods [13-15]. In addition to validating predicted genes, proteogenomics can be used to identify novel proteins, novel exons, novel translational start sites and protein isoforms based on the identification of novel splice junctions. Proteogenomic analysis has been previously employed to refine annotation of protein-coding genes in genomes of several organisms including human [16,17], *Drosophila*[18], *C. elegans*[19,20] and microorganisms (e.g. *Saccharomyces cerevisiae*[21,22], *Aspergillus niger*[23], *Mycobacterium tuberculosis*[24,25], *Candida glabrata*[26] and *Escherichia coli*[27]). In this study, we carried out an in-depth proteomic analysis of *C. neoformans* var. *grubii* to identify novel protein-coding regions in its genome in addition to the validation of predicted genes from this genome. For this, we subjected culture lysates of *C. neoformans* var. *grubii* to various fractionation methods followed by proteomic analysis on an LTQ-Orbitrap Velos mass spectrometer. We searched MS/MS data against protein database of *C. neoformans* var. *grubii,* which provided peptide evidence for 52% of the total annotated protein-coding genes. By searching MS/MS data against a six-frame translated genome database, we also identified 286 novel peptides with good spectral assignments that were unique to *C. neoformans* var. *grubii* genome. By corroborating these peptide sequences with ESTs and comparative genomics data, we identified 4 novel genes and revised the annotation of 104 gene structures in 111 instances. Finally, we also confirmed the annotated translational start sites of 524 proteins and identified 65 novel splice junctions.

## Results and discussion

To carry out a comprehensive analysis, we utilized a multipronged approach for protein/peptide fractionation including SDS-PAGE at the protein level and bRPLC and SCX at the peptide level. Seventy fractions (22 fractions from SDS-PAGE, 24 from bRPLC and 24 from SCX) were subjected to LC-MS/MS analysis. Overall, 485,714 MS/MS spectra obtained were searched against three databases, i) protein database containing sequences of 6,980 known/predicted proteins of *C. neoformans* var. *grubii* as reported in the genome database hosted by the Broad Institute (http://www.broadinstitute.org/annotation/genome/cryptococcus_neoformans/MultiHome.html); ii) six-frame translated genome database; and iii) N-acetylated peptide database.

### Proteomic evidence for predicted protein-coding genes in *C. neoformans* var. *grubii*

**Figure 1:**
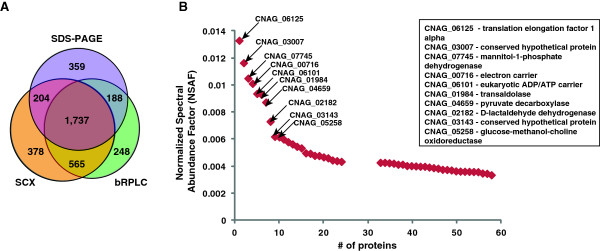
**Proteomics of *C. neoformans *var. *grubii*. A)** Venn diagram representing the number of proteins identified using different fractionation methods **B)** Scatter plot showing the normalized spectral abundance factor analysis of top 50 proteins identified in our study.

A search of the MS/MS data against the *C. neoformans* var. *grubii* protein database resulted in the identification of 184,030 peptide-spectrum matches (PSMs) that were filtered for first rank assignments and passed a 1% FDR cut-off. These PSMs resulted in the identification of 30,570 unique peptides, which corresponded to 3,674 proteins (52% of *C. neoformans* var. *grubii* proteome). Peptides and proteins identified in this study are provided as Additional file 1: Table S1 and Additional file 2: Table S2 respectively. Of these, multiple peptides supported the identification of 2,868 proteins, whereas 249 proteins are supported by a single peptide with more than one PSM for each peptide while 557 proteins are associated with a single peptide and a single PSM. We used multiple strategies for the isolation and fractionation of *C. neoformans* var. *grubii* proteome to increase the proteome coverage, which is evident with proteins exclusively identified in SDS-PAGE (359), SCX (378) and bRPLC (248) fractions (Figure 1A). We also obtained similar results in our previous proteogenomic analyses of *M. tuberculosis* and *C. glabrata*[24,26].

Genome annotation of *C. neoformans* var. *grubii* predicted 6,967 protein-coding genes from its nuclear genome and 13 protein-coding genes from its mitochondrial genome. Of the 6,980 annotated protein-coding genes, 2,200 genes were annotated based only on computational predictions and lacked any experimental evidence [6]. Our proteomic study has provided experimental validation, for the first time, for 746 protein-coding genes, covering 33% of genes which did not have any experimental evidence at the RNA or protein level. On the other hand, of the remaining 4,780 genes that had cDNA evidence, we identified 2,928 (61%) gene products. We observed a skewed detection ratio between computationally predicted genes (33%) and experimentally supported genes (61%), which was evident from global and unbiased approach used in this study, suggesting the fact that some of the computational predictions might not be true protein-coding genes. Rodrigues *et al*., have investigated the proteome of extracellular vesicles of *C. neoformans* and identified 76 proteins [28]. Using 2-DE and MALDI-TOF, Missall *et al.*, identified 32 proteins that were altered in response to nitric oxide stress in *C. neoformans*[29]. We have identified peptides belonging to 3,674 annotated protein-coding genes, which includes 10 proteins coded by mitochondrial genome. This constitutes ~50% coverage of the predicted protein-coding genes from the analysis of a single growth condition used in this study. The reason that we did not detect all of the proteins in this study is likely due to the fact that not all genes are expressed under a single growth condition (Loftus et al. subjected *C. neoformans* JEC21 (Serotype D) to 14 different growth conditions and obtained EST evidence for only 80% of protein-coding genes [5]) and that we did not identify proteins present at really low abundance. Another contributing factor could be that some of the computational predictions for protein-coding genes are false positives and thus cannot be experimentally verified.

Relative abundance of the identified cryptococcal proteins was determined by normalized spectral abundance factor. Proteins involved in translation (e.g. translation elongation factor 1 alpha) and metabolism (e.g. mannitol -1- phosphate dehydrogenase, transaldolase, pyruvate decarboxylase, D-lactaldehyde dehydrogenase) were among the most abundant proteins identified (Figure 1B and Additional file 2: Table S2).

### Genome search-specific peptides (GSSPs)

**Figure 2:**
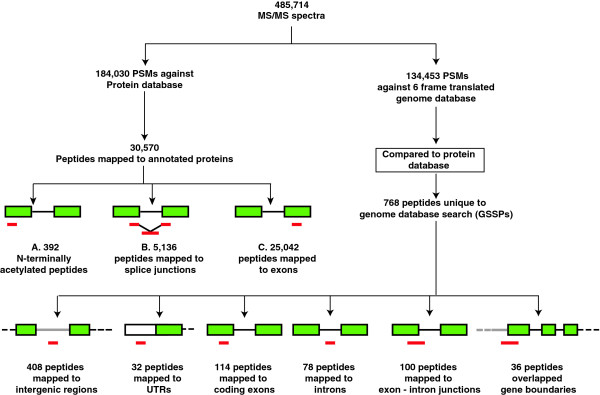
**Schematic workflow of proteogenomic analysis.** Mass spectrometry derived data was searched against protein database and six-frame translated genome database of *C. neoformans* var. *grubii*. Peptides mapping to the protein database confirmed annotated proteins and annotated splice junctions. C represents the number of peptides identified mapping to exons excluding A and B. Peptides unique to six-frame translated genome database were categorized based on their mapping to intergenic regions and regions within the annotated genes. These peptides were used to refine the annotation of genome.

GSSPs are those peptides, which are identified from the search of MS/MS data against six-frame translated genome database but are not represented in the known protein databases. In our study, search of MS/MS spectra against six-frame translated genome database resulted in identification of 134,453 PSMs corresponding to 22,377 peptides. Among these, 768 novel peptides did not belong to any of the known proteins in *C. neoformans* var. *grubii*. Of the 768 novel peptides unique to genome database search, 286 peptides passed manual validation for good spectral assignments. Those peptides that passed manual validation were the only ones considered for further analysis. We also discarded any peptides that had multiple hits in the genome or were post-translationally modified. Of the 286 peptides analyzed for gene models based on orthology-based evidence and gene prediction models, 144 peptides contributed to identification of 4 novel genes and 111 revised gene models (Figure 2 and Table 1).

**Table 1:** **Summary of novel findings in proteogenomic analysis of *C. neoformans *** **var. *grubii***

**Categories**	**Novel identifications/refinement of gene models**
Novel genes	4
Novel exons	17
Refinement of gene boundaries	19
Refinement of exon boundaries	53
Protein coding evidence in UTRs	13
Correction of translational frame	8
Novel protein start site	1

Since use of hypothetical databases such as 6-frame translated genome database for peptide identification might, in theory, increase the chance of false identifications, we plotted the score distribution of peptides from protein database search and the genome search-specific peptides (from six frame translation search) and found that the score was indeed lower for the GSSPs (Additional file 3: Figure S1) [30]. This is in agreement with our finding that only 286 out of 768 GSSPs passed our manual verification. Overall, we believe that our approach is a conservative approach and that we might have a number of false negatives because of technical reasons (FDR threshold, no modifications allowed, no ionizable peptides in the detection range, only unique hits considered, only one missed cleavage allowed) as well as biological ones (exon-exon spanning peptides will be missed). We carried out RT-PCR for 50 gene models and were able to validate 47 novel genes and revised gene models (see details below). We believe that we were able to achieve an overall success rate of 94% for these validation studies because of the conservative approach adopted in our study.

### Identification of novel protein-coding genes

**Figure 3:**
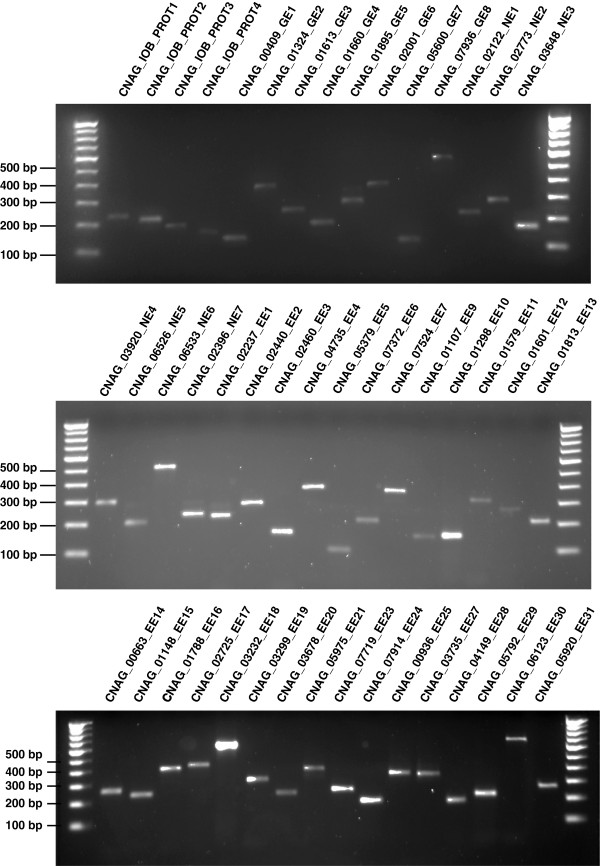
**RT-PCR based validation of novel and revised gene models.** RT-PCR validation was carried out for 47 novel and revised gene models. Transcript identifier for each gene model is indicated above each lane. These RT-PCR products were sequenced and submitted to GenBank.

**Figure 4:**
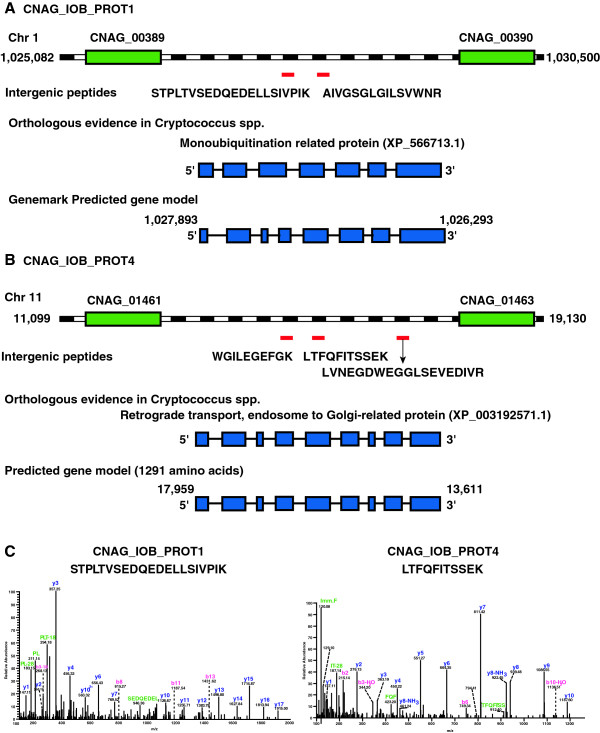
**Identification of novel protein-coding genes based on peptides mapping to intergenic regions. A)** GSSP mapped to intergenic region in Chromosome 1 between CNAG_00389 and CNAG_00390. Gene prediction programs predicted 358 amino acids long proteins from this region, which is homologous to monoubiquitination protein of *C. neoformans* var. *neoformans.***B)** Three peptides mapped to intergenic region between CNAG_01461 and CNAG_01463. Gene prediction programs predicted protein-coding gene in this region. The predicted protein has homology to retrograde transport, endosome to Golgi related protein, XP_776934.1 (*C. neoformans* var. *neoformans* B-3501A), XP_569839.1 (*C. neoformans* var*. neoformans* JEC21), XP_003192571.1 (*C. gattii* WM276). **C)** The MS/MS spectra of intergenic peptides STPLTVSEDQEDELLSIVPIK and LTFQFITSSEK are shown.

Novel protein-coding genes in the genome were identified using GSSPs that mapped to intergenic regions. We propose 4 novel gene models with additional evidence in the form of orthology and ESTs (Table 2). Subsequently, these novel genes were also validated by cDNA sequencing (Figure 3). Novel protein 1 (CNAG_IOB_PROT1) of length 358 amino acids was identified based on two GSSPs that mapped to the intergenic region between CNAG_00389 and CNAG_00390 genes located on chromosome 1. This novel protein was found to be homologous to monoubiquitination related protein of *Cryptococcus neoformans* var*. neoformans* JEC21 (XP_566713.1). The second novel protein was identified based on two GSSPs DLEIGAPVSIGDTPSPEDK and SLSPEVQASLPADIR, which mapped to the intergenic region between CNAG_03798 and CNAG_03805 genes on chromosome 2. Analysis of this intergenic region using alternate gene prediction programs revealed a novel protein-coding region (CNAG_IOB_PROT2) homologous to integrase rve protein of *C. neoformans* var. *neoformans* B-3501A (XP_777044.1). The third novel protein-coding gene was identified in an intergenic region on chromosome 5 based on a single GSSP between CNAG_07399 and CNAG_06854 genes. BLASTX analysis of this intergenic region revealed a novel open reading frame (CNAG_IOB_PROT3) of 355 amino acids. This novel protein is similar to a hypothetical protein in *C. neoformans* var*. neoformans* JEC21 (XP_570553.1). Three GSSPs, WGILEGEFGK, LTFQFITSSEK and LVNEGDWEGGLSEVEDIVR, were clustered in the intergenic region between CNAG_01461 and CNAG_01463 genes on chromosome 11. Analysis of this intergenic region revealed a novel protein-coding region (CNAG_IOB_PROT4) encoding a 1,292 amino acid residues long protein. This novel protein was found to be homologous to retrograde transport, endosome to Golgi-related protein in *C. neoformans* var. *neoformans* (Serotype D and Serotype AD) and *C. gattii* (Serotype B and C). Illustration of novel genes CNAG_IOB_PROT1 and CNAG_IOB_PROT4 is provided in Figure 4. MS/MS spectra of novel peptides belonging to these novel genes are shown in Additional file 4: Figure S2.

**Table 2:** **List of novel proteins identified in *C. neoformans *** **var. *grubii***

	**Protein name**	**Genome search specific peptide**	**Genomic co – ordinates of novel gene**	**GenBank Accession (Transcript identifier)**	**Homologs in related species**	**Putative function**
1	CNAG_IOB_prot1	STPLTVSEDQEDELLSIVPIK; AIVGSGLGILSVWNR	Chromosome 1: 1026293–1027893; Negative strand	GenBank: JZ152657.1 (CNAG_IOB_PROT1)	XP_777889.1 (*Cryptococcus neoformans* var. *neoformans* B-3501A); XP_566713.1 (*Cryptococcus neoformans* var*. neoformans* JEC21); EAU84048.2 (*Coprinopsis cinerea okayama*7#130); EDP43708.1 (*Malassezia globosa* CBS 7966);	Monoubiquitination related protein
2	CNAG_IOB_prot2	DLEIGAPVSIGDTPSPEDK; SLSPEVQASLPADIR	Chromosome 2: 852949–855029; Positive strand	GenBank: JZ152658.1 (CNAG_IOB_PROT2)	XP_777044.1 (*Cryptococcus neoformans* var. *neoformans* B-3501A);	Integrase rve protein
3	CNAG_IOB_prot3	LFDNDADGDDDDDQGAVNVNIR	Chromosome 5: 65512–66695; Positive strand	GenBank: JZ152659.1 (CNAG_IOB_PROT3)	XP_570553.1 (*Cryptococcus neoformans* var*. neoformans* JEC21); XP_776190.1 (*Cryptococcus neoformans* var. *neoformans* B-3501A);	Hypothetical protein
4	CNAG_IOB_prot4	WGILEGEFGK, LTFQFITSSEK, LVNEGDWEGGLSEVEDIVR	Chr 11: 13611–17959; Negative strand	GenBank: JZ152660.1 (CNAG_IOB_PROT4)	XP_776934.1 (*Cryptococcus neoformans* var. *neoformans* B-3501A), XP_569839.1 (*Cryptococcus neoformans* var*. neoformans* JEC21), XP_003192571.1 (*Cryptococcus gattii* WM276)	Retrograde transport, endosome to Golgi-related protein

### Confirmation and correction of splice junctions

*C. neoformans* genome has a relatively complex gene structure with an average of 5 short introns per gene of average size of ~67 bases. Determination of exon-boundaries in such short intron-rich genome is known to be challenging [5]. Therefore, accuracy of splice sites proposed by gene prediction programs can be verified using proteomic evidence in the form of splice-junction peptides. Peptides encompassing these splice junctions would be useful in verifying the splice sites or in annotating the existence of novel ones [10]. Our proteomic approach enabled us to identify 5,136 peptides mapping to exon–exon junctions from 1,919 annotated genes. This led to validation of 3,863 splice sites in *C. neoformans* var. *grubii* genome and simultaneously validated short introns predicted from computational prediction pipelines. We have provided a list of these splice junction peptides in Additional file 5: Table S3.

In addition to validating predicted exon-exon boundaries in the intron rich genome of *C. neoformans* var. *grubii,* we also identified novel exons and refined the structures of several annotated exons. GSSPs which overlapped exons and associated intronic regions aided in the identification of novel exons and also in revising the splice junctions for the corresponding genes. Seventeen novel exons were identified using 20 unique peptides mapping to intronic regions (Table 3). Of these, 7 novel exons were validated by RT-PCR and confirmed by cDNA sequencing (Figure 3; Table 3). Introns following these 17 novel exons were found to have the canonical splicing signals [10].

**Table 3:** **List of novel exons identified using peptide evidence in *C. neoformans *** **var. *grubii***

	**Gene ID**	**Genome search specific peptide**	**Novel exon co – ordinates**	**GenBank Accession (Transcript identifier)**	**Orthologous evidence for novel exons identified by GSSPs**
1	CNAG_02122	AcALLEGLESGLSR	Chr 6: 1136454 – 1136507; Negative	GenBank: JZ152661.1 (CNAG_02122_NE1)	XP_571070.1 (*Cryptococcus neoformans* var. *neoformans* JEC21); XP_003194441.1 (*Cryptococcus gattii* WM276); EIW67330.1 (*Tremella mesenterica* DSM 1558)
2	CNAG_02773	TAITIKPALAAQAGK	Chr 3: 801827 – 801913; Negative	GenBank: JZ152662.1 (CNAG_02773_NE2)	XP_003192989.1 (*Cryptococcus gattii* WM276); XP_570007.1 (*Cryptococcus neoformans* var. *neoformans* JEC21)
3	CNAG_03648	VIFVDADQIVR	Chr 2: 438306 – 438539; Positive	GenBank: JZ152663.1 (CNAG_03648_NE3)	XP_777193.1 (*Cryptococcus neoformans* var. *neoformans* B-3501A); XP_388116.1 (*Gibberella zeae* PH-1); XP_001832620.2 (*Coprinopsis cinerea okayama*7#130);
4	CNAG_03920	YYDLGMESR	Chr 2: 1190442 – 1190507; Positive	GenBank: JZ152664.1 (CNAG_03920_NE4)	XP_569234.1 (*Cryptococcus neoformans* var. *neoformans* JEC21); XP_003191314.1 (*Cryptococcus gattii* WM276)
5	CNAG_06526	TYPLALDLWDSGSSVIIR; IASFPLNFISR	Chr 7: 12832–13094; Positive	GenBank: JZ152665.1 (CNAG_06526_NE5)	XP_567229.1 (*Cryptococcus neoformans* var. *neoformans* JEC21)
6	CNAG_06533	SNGYYSPITYFLAK; LLFDIIPLR	Chr 7: 36868 – 37134; Positive	GenBank: JZ152666.1 (CNAG_06533_NE6)	XP_571629.1 (*Cryptococcus neoformans* var. *neoformans* JEC21); XP_003194552.1 (*Cryptococcus gattii* WM276); XP_757847.1 (*Ustilago maydis* 521); XP_750621.1 (*Aspergillus fumigatus* Af293)
7	CNAG_02396	FNGPVDFER	Chr 6: 435584 – 435658; Positive	GenBank: JZ152667.1 (CNAG_02396_NE7)	XP_570777.1 (*Cryptococcus neoformans* var*. neoformans* JEC21); XP_003194186.1 (*Cryptococcus gattii* WM276)
8	CNAG_00853	TERYPLTLGR	Chr 1: 2257775 – 2257895; Positive	-	XP_567069.1(*Cryptococcus neoformans* var. *neoformans* JEC21), XP_777691.1 (*Cryptococcus neoformans* var. *neoformans* B-3501A), XP_003191768.1 (*Cryptococcus gattii* WM276)
9	CNAG_07863	AELEMLVQR	Chr 10: 1004163 – 1004219; Negative	-	XP_567270.1 (*Cryptococcus neoformans* var. *neoformans* JEC21), XP_773061.1 (*Cryptococcus neoformans* var. *neoformans* B-3501A), XP_003193980.1 (*Cryptococcus gattii* WM276)
10	CNAG_01261	VTGQSPEDEDWLVGETLDGSHAGGFPK	Chr 5: 763635 – 763722; Negative	-	XP_570166.1 (*Cryptococcus neoformans* var. *neoformans* JEC21)
11	CNAG_02407	GGASIFEMVR; GGASIFEmVR	Chr 6: 401719 – 401783; Negative	-	XP_775427.1 (*Cryptococcus neoformans* var*. neoformans* B-3501A);
12	CNAG_02292	YDIDLENKR	Chr 6: 684574 – 684630; Positive	-	XP_775540.1 (*Cryptococcus neoformans* var*. neoformans* B-3501A);
13	CNAG_02230	FIDPAESGAVIPILHVNGYK	Chr 6: 853119 – 853178; Negative	-	XP_003194389.1 (*Cryptococcus gattii* WM276); XP_775249.1 (*Cryptococcus neoformans* var*. neoformans* B-3501A); XP_570860.1 (*Cryptococcus neoformans* var. *neoformans* JEC21)
14	CNAG_02147	LLLRPIWKPR	Chr 6: 1080395 – 1080490; Positive	-	XP_775114.1 (*Cryptococcus neoformans* var*. neoformans* B-3501A)
15	CNAG_03648	ASSVVTAAYKPLDGEGIFAPAQSTR	Chr 2: 436700–436837; Positive	-	XP_568822.1 (*Cryptococcus neoformans* var. *neoformans* JEC21);XP_003192380.1 (*Cryptococcus gattii* WM276)
16	CNAG_04955	GAVLVNVGR	Chr 4: 73400–73459; Negative	-	XP_568183.1 (*Cryptococcus neoformans* var. *neoformans* JEC21); XP_773659.1 (*Cryptococcus neoformans* var. *neoformans* B-3501A); XP_003193297.1 (*Cryptococcus gattii* WM276)
17	CNAG_05059	GLLDKFGEDR	Chr 4: 323430–323477; Positive	-	XP_568096.1 (*Cryptococcus neoformans* var. *neoformans* JEC21); EIW71449.1 (*Tremella mesenterica* DSM 1558)

We found 53 examples of exon extensions, thereby revising the corresponding predicted gene models (Additional file 6: Table S4). These exon extensions were also supported by the presence of similar sequences in orthologous genes. In addition, we validated a subset of these exon extensions by cDNA sequencing (Figure 3). Of the 53 cases of exon extension, splice donor site was revised in 37 cases while the splice acceptor site was revised in remaining 16 cases. These altered splice acceptor and donor sites had the canonical splice acceptor sites – 5′GU and 5′ GC and splice donor site – 3′AG [10]. Additional file 7: Figure S3 shows an example of exon extension through identification of peptides mapping to intron and exon–intron junction of CNAG_02460. The current annotation of CNAG_02460 comprises 4 exons. We identified 19 peptides mapping to first and second exon of CNAG_02460. From GSSP analysis, we found one peptide mapping to junction of second exon and second intron and two peptides mapping to the second intron. These peptides supported a model in which the two exons could be merged. We validated this exon extension by RT-PCR and cDNA sequencing (Figure 3).

### Refinement of coding DNA sequence coordinates

Coding DNA Sequence (CDS), the portion of genomic DNA sequence composed of exons that is translated into protein, is often delineated by gene prediction algorithms. Incorrect prediction of translational start sites and stop codons are frequently encountered in *ab initio* predicted gene models. Peptides mapping to upstream and downstream of genes suggest changes to CDS boundaries of genes. These peptides alter the annotated translational start sites or stop codons resulting in the extension of annotated genes either towards N-terminus or C-terminus of proteins, respectively. In this study, we extended N-termini of 14 proteins using 18 GSSPs as evidence; and C-termini of 5 proteins using 5 GSSPs mapping either to intergenic region or junction of intergenic region and CDS (Additional file 8: Table S5). N-terminal extension of CNAG_05600 protein with a peptide mapping upstream of the gene is depicted in Additional file 9: Figure S4. The peptide extended the CNAG_05600 protein, which belongs to indigoidine synthase A family, from 707 amino acids to 773 amino acids. The newly extended part of this gene is found to be conserved in other serotypes of *Cryptococcus*. Analysis of another intergenic peptide, which mapped downstream of gene CNAG_00409, using alternate gene prediction programs extended the C-terminus of protein. The revised protein has an orthologous protein in *C. neoformans* var. *neoformans* B-3501A (XP_777909.1) and *C. neoformans* var. *neoformans* JEC21 (XP_566744.1). We have validated these gene extensions by RT-PCR and submitted sequences of these cDNAs to GenBank (Figure 3).

In another type of refinement of CDS co-ordinates using GSSPs mapping to UTRs, we found 8 instances of N-terminal in-frame extensions using peptides that mapped to 5′ UTR of genes and 5 cases of C-terminal extensions with the aid of peptides mapping to 3′ UTR of genes (Additional file 10: Table S6). For instance, CNAG_01159 gene was annotated to code for 466 amino acids long pre-mRNA splicing factor SLU7 protein. We identified a peptide in 5′ UTR of the gene and were able to extend the protein at N-terminus resulting in a 586 amino acids long protein, which is conserved in *C. neoformans* var. *neoformans, Coprinopsis cinerea okayama* and *Ustilago maydis*. We also extended C-terminus of CNAG_00768 protein to 368 amino acids, which was annotated to be 164 amino acids long, by finding 4 peptides mapping to 3′ UTR of its gene (Additional file 11: Figure S5). The extended protein was found to be conserved in *C. neoformans* var. *neoformans* and *C. gattii.*

### Alternate frame of translation

Identification of the correct reading frame is essential to confirm the translational read out of hypothetical proteins. Erroneous annotation of reading frames in hypothetical proteins is possible as the start codons are only putative translational initiation sites [31]. Peptide-based evidence provides unique advantage of confirmation of translational frame of the annotated gene. MS/MS data search against six-frame translated genome database identified 10 exonic peptides in 8 genes, which did not match the annotated frame of translation of the corresponding gene. These 10 peptides also had orthologous evidence in other serotypes of *Cryptococcus*, which indicates that the annotated translational frame needs to be revised in these exonic regions of the genes. We were able to determine the correct reading frame by using peptide evidence. We identified 3 GSSPs mapping to coding exon 11 and exon 12 of CNAG_05480, the gene coding for hexokinase, but in a different frame of translation. MS/MS spectra and details of exonic peptides that altered the translational frame can be found in Additional file 12: Figure S6 and Additional file 13: Table S7.

### N-terminally acetylated peptide-based confirmation of translational start sites

**Figure 5:**
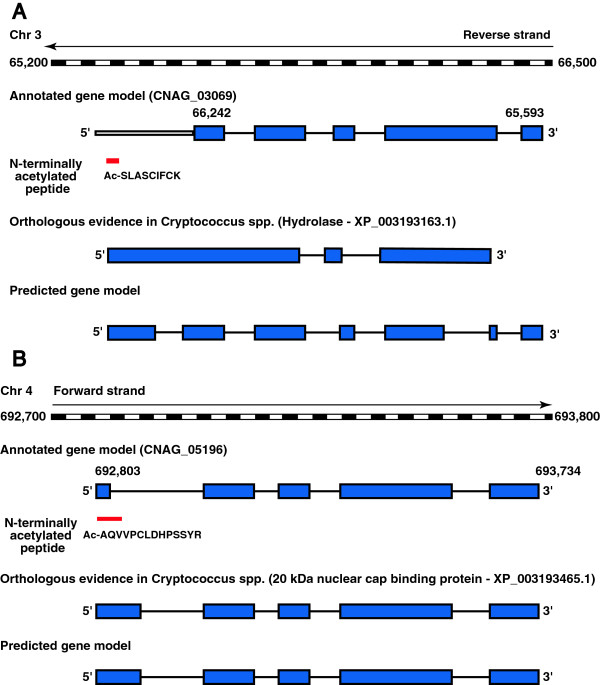
**Identification of protein start site using N-terminally acetylated peptide. A)** N-terminally acetylated peptide Ac-SLAScIFcK identified upstream to CNAG_03069 corrected the annotated protein start site. **B)** N-terminally acetylated peptide Ac- AQVVPCLDHPSSYR found overlapping exon and intron junction confirmed the annotated protein start site and extended the first exon of CNAG_05196.

Conventionally, protein start sites have been assigned based on the longest open reading frame and homology-based comparative genomics [32]. Most eukaryotic proteins are acetylated at their N-termini, usually after the removal of the initiator methionine. Most often, N-terminal methionine is cleaved when the second amino acid residue is any of the following amino acids – Gly, Ala, Ser, Thr, Cys, Pro or Val. N-terminal Met is generally retained when the second amino acid is Asp, Glu or Asn [33]. In some cases, up to 3 amino acid residues can be removed from the N-terminal end. Identifying N-terminal acetylated peptides by mass spectrometry is an excellent method for determining the translational start sites of proteins [34]. Identification of N-terminally acetylated peptides and N-terminally semi-tryptic peptides can be used to determine the translational start sites [24,35]. In *C. neoformans* var. *grubii,* we identified 392 N-terminally acetylated peptides and 277 peptides with up to 2 amino acids cleaved from N-terminal end of protein, confirming the annotated translational start sites of 524 proteins. In 296 proteins, translational start sites were confirmed with N-terminally acetylated peptides, in 195 proteins, it was confirmed with identification of non-acetylated N-terminal peptides. In cases of another set of 33 proteins, both types of peptides were found. Of 392 N-terminally acetylated peptides, 104 peptides had modified alanine residue and 209 peptides had acetylated serine, which is in agreement with similar findings in most of previous the investigations on N-terminally acetylated peptides [36]. In addition, we also identified 2 GSSPs that were acetylated at the N-termini using our in-house developed custom N-terminal database to identify novel start sites of proteins. One of the N-terminally acetylated peptide Ac-SLASCIFCK that mapped upstream to hydrolase gene (CNAG_03069) extended the protein at its N-terminus (Figure 5A). This revised and extended protein sequence is supported by homologous protein in *C. neoformans* var. *neoformans* (XP_569386.1) and *C. gattii* (XP_003193163.1)*.* Interestingly, the second N-terminally acetylated GSSP Ac- AQVVPCLDHPSSYR, besides confirming the annotated protein start site of 20 kDa nuclear cap binding protein, CNAG_05196; also extended the 3′ end of exon 1 into the annotated intron thereby modifying this gene model (Figure 5B). The modified gene structure was supported by the presence of similar proteins in *C. neoformans* var. *neoformans* B-3501A (XP_773409.1) and *C. gattii* WM276 (XP_003193465.1).

## Conclusions

Opportunistic fungal infections are emerging as a serious threat to human health. Therapeutic interventions to manage these infections is difficult as many antifungals have severe side effects [37]. Global molecular profiling of fungal pathogens will provide a platform for subsequent comparative genomic and proteomic analyses to identify molecules that are associated with virulence and pathogenesis. In this study, we carried out in-depth proteomic profiling of *C. neoformans* var. *grubii,* the most virulent form among *C. neoformans* variants. We have provided protein-coding evidence for 52% of annotated genes from *C. neoformans* var. *grubii.* In addition, we also identified a large number of novel protein-coding regions, which are not represented in the current *C. neoformans* var. *grubii* protein database. Genome of *Cryptococcus*, rich in short introns, is being explored as a model to understand evolutionarily conserved mechanisms of splicing [38]. In this study, besides confirming >3,800 annotated splice junctions, proteogenomics helped us identify several novel splice junctions and revise a number of exon-intron junctions. Similar high-resolution mass spectrometry based investigation of proteome of the pathogen under different biological and experimental conditions would provide differential protein expression data, which will facilitate our understanding of protein-level changes associated with pathogenesis.

## Methods

### Strain and growth conditions

*C. neoformans* var. *grubii* culture (MTCC 1353), was obtained from Microbial Type Culture Collection and Gene Bank resource, Chandigarh in India. The microbe was cultured in Sabouraud’s Dextrose broth at 37°C until the cells reached log phase (OD600 = 0.6). Approximately, 5 × 10^9^ cells were harvested from one litre culture after centrifugation. The cell pellets were washed ten times using phosphate buffered saline.

### Protein isolation

The cell pellets were resuspended in 8 M urea and were then subjected to disruption using glass beads in a cell disruptor (Disruptor Genei SI-D267, Scientific Industries Inc. NY) for 30 min at 4°C. Cell lysates were further homogenized by sonication in an ice bath for three 30 sec cycles with 1 min intervals, using an ultra sonicator (Microson XL, NY). The samples were then centrifuged at 10,000 × g for 10 min at 4°C to obtain supernatant. Protein estimation was carried out using Lowry’s assay [39]. Protein isolated in 8 M urea was used for in solution digestion followed by peptide fractionation using strong cation exchange (SCX) chromatography and basic pH reversed-phase liquid chromatography (bRPLC).

For SDS-PAGE, cell pellets were lyophilized overnight and then ground in liquid nitrogen using mortar and pestle to obtain a fine powder as described by Crestani *et al*. [40]. Samples were then suspended in the buffer containing 50 mM Tris–HCl, pH 7.5, 1 mM EDTA, 1 mM PMSF. Protein lysates were vortexed for 5 min and then centrifuged at high speed for 20 min. Supernatant was collected. Cell debris was resuspended in the above-mentioned buffer, vortexed for 5 min and sonicated (three 30 sec cycles with 1 min interval in an icebath). Supernatant collected after centrifugation was pooled with the first supernatant. Protein estimation was carried out using Lowry’s assay.

### Trypsin digestion and protein/peptide fractionation

For SDS-PAGE, 250 μg of the protein lysate was resolved using 12% gel and stained with Coomassie blue stain. The lane was divided into 22 bands and these were subjected to in-gel trypsin digestion as described earlier [41]. The peptides extracted were dried and subjected to LC-MS/MS analysis. For peptide level fractionation, in solution digestion was carried out as described previously [41]. Briefly, reduction was carried out by incubating 500 μg of total protein lysate with 5 mM Dithiothreitol (DTT) at 65°C for 45 min and was then alkylated with 20 mM iodoacetamide at room temperature for 10 min to irreversibly modify cysteine. The modified protein lysate was subjected to trypsin digestion at 37°C overnight, with an enzyme: substrate ratio of 1:20. The digest was then desalted using Sep-Pak C18 columns (Waters Corporation, Milford, MA) and lyophilized at -52°C. Subsequently, the sample was split into two equal halves and fractionated by SCX [42] and bRPLC [43].

SCX fractionation was carried out on a PolySulfoethyl A column (PolyLC, Columbia, MD; 200 Å, 5 μm, 200 × 2.1 mm) using an Agilent 1200 series HPLC system containing a binary pump, autosampler, UV detector and a fraction collector. Phosphoric acid was added to in-solution digests to adjust its pH to 2.8 and then diluted to 1 mL using SCX solvent A (10 mM potassium phosphate buffer in 20% ACN, pH 2.8). Fractionation of peptides (0.2 mL fractions) was carried out by a linear gradient of solvent B (10 mM KH2PO4, 350 mM KCl, 20% acetonitrile, pH 2.8) for 70 min. The fractions were completely dried, reconstituted in 40 μL of 0.1% TFA, desalted using C_18_ StageTips and subjected to LC - MS/MS analysis. bRPLC was performed on XBridge C_18_, 5 μm 250 × 4.6 mm analytical column (Waters Corporation, Milford, MA) with a flow rate of 1 mL/min using an Agilent 1200 series HPLC system. The mobile phase A consisted of 7 mM TEABC in water and B consisted of 7 mM TEABC in 90% Acetonitrile. Sample separation was accomplished using the following gradient: 1% B for 0–5 min, 10% B for 5–10 min, 35% B for 10–40 min and 100% B for 40–45 min. Ninety six fractions were collected in 96 well plate containing 1% formic acid. The fractions were dried to half the original volume and were concatenated into 24 fractions by combining 1, 13, 25 and 37; 2, 14, 26 and 38 and so on. These 24 fractions were subjected to LC-MS/MS analysis.

### LC-MS/MS analysis

Nanoflow electrospray ionization tandem mass spectrometric analysis of peptide samples was carried out using LTQ-Orbitrap Velos mass spectrometer (Thermo Scientific, Bremen, Germany) interfaced with Easy-nLC (Thermo Scientific, Bremen, Germany). The chromatographic capillary columns used were packed in-house with Magic C_18_ AQ (Michrom Bioresources, Inc., Auburn, CA, USA) (5 μm particle size, pore size 100 Å) reversed phase material in 100% acetonitrile at a pressure of 1000 psi. The peptides sample from each fraction was enriched on a pre-column (75 μm × 2 cm) at a flow rate of 5 μL/min with solvent A (0.1% formic acid in water). Peptides were separated on an analytical column (75 μm × 10 cm) at a flow rate of 350 nL/min using a linear gradient of 7% to 30% solvent B (0.1% formic acid in 95% acetonitrile) over 60 minutes. Mass spectrometry analysis was carried out in a data dependent manner with full scans within 350–1800 m/z acquired using an Orbitrap mass analyzer at a mass resolution of 60,000 at 400 m/z. For each duty cycle, twenty most intense precursor ions from a survey scan were selected for MS/MS and detected at a mass resolution of 15,000 at m/z of 400, also in an Orbitrap analyzer. The fragmentation was carried out using higher-energy collision dissociation (HCD) with 39% normalized collision energy. Dynamic exclusion was set for 30 seconds with a 10 ppm mass window. The automatic gain control for full FT MS was set to 0.5 million ions and for FT MS/MS was set to 0.1 million ions with a maximum ion injection times of 100 ms and 200 ms, respectively. Internal calibration was done using lock-mass from ambient air (m/z 445.1200025) as described previously [44]. Other parameters include spray voltage of 2.0 kV, capillary voltage of 250. The raw data obtained was submitted to ProteomeCommons (https://www.proteomecommons.org/).

### Database searches for peptide identification

Proteome Discoverer version 1.3 (Thermo Scientific) platform integrated with Sequest and Mascot search engines was used to search the mass spectrometry data against protein and other customized databases. The protein database consisted of 6,980 protein sequences, which included protein sequences encoded by genome (6,967) and mitochondrial genome (13) of *C. neoformans* var. *grubii* downloaded from the genome database at Broad Institute (gene set version 4.0) (http://www.broadinstitute.org/annotation/genome/cryptococcus_neoformans/MultiHome.html) [6] and common contaminants. Carbamidomethylation of cysteine was used as fixed modification whereas oxidation of methionine and protein N-terminal acetylation were used as variable modifications. A maximum of one missed cleavage, mass deviation of 20 ppm and 0.1 Da were allowed for MS and MS/MS, respectively. False discovery rate of 1%, as calculated by enabling the peptide sequence analysis using decoy database, was used as a cut-off value for reporting identified peptides. The peptide and protein data were extracted using high peptide confidence and top one peptide rank filters. Relative abundance of proteins in *C. neoformans* var. *grubii* was determined by calculating normalized spectral abundance factors (NSAF) for each protein identified in the study as previously described [45]. NSAF for a protein k was calculated as dividing the total number of peptide spectral matches (S) identified for protein k by protein length (L) and then divided by the sum of S/L ratio for all proteins.

### Workflow for genome annotation

The whole genome sequence of *Cryptococcus neoformans* var. *grubii* was downloaded from Broad Institute’s website and translated into six reading frames. These translated sequences were fetched as amino acid sequences from stop codon to the next stop codon, stored in a database as a six-frame translated genome database. The peptide data obtained from MS/MS data searches against six-frame translated genome database were compared with protein database to find unique peptides, which are not represented in proteins database. These peptides were referred to as “genome search specific peptides (GSSPs).” GSSPs were mapped on to the genome by tblastn and the genomic co-ordinates of these peptides were fetched. Only those GSSPs which mapped to single locus in the genome were considered for further analysis. Based on the genomic regions they mapped to, these GSSPs were classified as follows: (i) peptides mapping to intergenic regions; (ii) peptides mapping to UTRs; (iii) peptides mapping to introns; (iv) peptides mapping to junctions of exon–intron; (v) peptides mapping to coding exons but translated in a different frame; and (vi) peptides mapping onto gene boundaries. Peptides that overlapped with annotated gene models were used to correct the existing gene annotation. The intergenic peptides were analyzed to identify novel protein-coding regions. Comparative genome analysis of the genomic region flanking GSSPs and/or alternative gene models by various prediction programs – Augustus [46], Geneid [47], GLEAN [48], GeneMark [49] and Twinscan [50], which were incorporated in the Broad Institute’s genome browser were used to determine the novel gene models.

Most eukaryotic proteins are known to be N-terminally modified. Translational start sites can be determined by identifying N-terminally acetylated peptides [35,51]. Since we allowed identification of only tryptic peptides from genome database, novel protein N-terminal peptides could not be identified as they appear as semitryptic peptides in the six frame translated genome sequences. Hence, from the six-frame translated genome database, we created a separate database of potential N-terminal peptides starting with methionine and ending with either lysine or arginine with sequence length from 7 amino acids to 15 amino acids. MS/MS data was searched against this database using Mascot search engine, while defining variable modification of protein N-terminal acetylation. As with Mascot search engine it is possible to identify protein N-terminal peptides with or without cleaved initiator methionine, we could identify peptides with N-terminal acetylation at second amino acid or initiator methionine.

### Validation of gene models by RT-PCR

A subset of novel or revised gene models was validated using RT-PCR followed by cDNA sequencing. Gene model specific primers were designed using Primer3 software [52]. The primers were designed to span the revised regions and designed across exons. Total RNA was isolated from mid log phase culture of *C. neoformans* var. *grubii* as described by Yang *et al.*[53]. Briefly, cell pellet obtained from 50 mL culture was washed 10 times with ice cold PBS and lyophilized overnight. The pellets were ground using liquid nitrogen and suspended in 1 mL Qiazol (Catalog No. 79306, Qiagen, Valencia, CA). The suspension was brought to room temperature. To the suspension, 200 μL of chloroform was added and mixed well. The tubes were then incubated at room temperature for 10 min and later centrifuged at high speed for 15 min at 4°C. The upper aqueous phase was separated and collected in a fresh tube. RNA was precipitated with 500 μL of isopropanol by incubating at room temperature for 15 min followed by centrifugation at high speed for 15 min at 4°C. The pellet was washed with 70% ethanol and dried. The pellet was suspended in 50 μL of RNase free water. RNA was then subjected to DNase treatment to remove any genomic DNA using RNeasy mini kit (Catalog No. 74104, Qiagen, Valencia, CA) following manufacturer’s instructions. cDNA synthesis was carried out with about 1 μg of total RNA using QuantiTect Reverse Transcription kit (Catalog No. 205311, Qiagen, Valencia, CA). PCR was carried out using 1 μL of cDNA, 0.1 μM of each forward and reverse primers, 1.5 mM MgCl_2_ 0.2 mM dNTP mix, 1.5U of Taq polymerase and PCR buffer in 50 μl reaction volume. Thermal cycling conditions performed to amplify the target sequence comprised initial denaturation cycle of 95°C for 3 min, 40 cycles of amplification with 95°C for 15 sec, 55°C for 60 sec and 72°C for 30 sec. The list of forward and reverse primers used for validation is provided in Additional file 14: Table S8. PCR carried out with RNA served as negative control. Amplicon sizes were determined by running 5 μL of reaction on 1.5% agarose gel along with 100 bp DNA ladder. PCR products were subjected to PCR clean up using Qiaquick PCR purification kit (Catalog No. 28104, Qiagen, Valencia, CA). The purified products were sequenced using Applied Biosystems 3730xl DNA analyzer Big Dye Terminator. cDNA sequences thus obtained were submitted to GenBank.

### Data availability

The data associated with this manuscript can be downloaded from the ProteomeCommons.org Tranche network using the following hash:

Set 1

m9ScGjS + 0D/0f6p4IyDJs + cRZCggBOwvURhehaC7cNG + IPFQbtXtUV7eJ2wIeMlzyw2EhePB1kvIasad5RBmWbbVR00AAAAAAAALYw==

Set 2

4fXMiR4I/ZZlQyipQcLnCrPrAXFvRe8gC5Jcz9orBnVq9yZclXD81FmjviuphY + A1oBFb9lkKLqpPdU + H0YeOjyDJ2EAAAAAAAAKzA==

Set 3

uf + UIAxU1l93cNDStQLjwt6ONgjPSYSs4sWMBP3i/kiCABvEos5tWIu78pS06fBT2GJklhuZDQ/Ro0muhtAfNvah1SAAAAAAAAALTw==

## Abbreviations

GSSP: Genome search specific peptide; SCX: Strong cation exchange; bRPLC: Basic pH reversed-phase liquid chromatography; CDS: Coding DNA sequence; PSM: Peptide spectrum matches; TEABC: Triethyl ammonium bicarbonate; MS/MS: Tandem mass spectrometry.

## Competing interests

The authors declare that they have no competing interests.

## Authors’ contributions

TSKP, HG, AP and RR conceived the study and designed the experiments; LDNS, RSN, DSK, VNP and JEK carried out the experiments; LDNS, DSK, RSN, BM, TS, NS, AR, AKM and PK analyzed the data; LDNS, DSK, TS, TSKP and AP wrote the manuscript; BN, AC, RR, AP, HG and TSKP provided critical comments. All authors read and approved the final manuscript.

## Supplementary Material

Additional file 1: Table S1 List of unique peptide sequences identified in *C. neoformans*. Click here for file

Additional file 2: Table S2 List of proteins identified in *C. neoformans*. Click here for file

Additional file 3: Figure S1 Search score distributions for the peptides identified in the study A) Mascot Score, B) Sequest Score distribution for peptides identified from protein database search, C) Mascot Score, D) Sequest Score distribution for GSSPs, E) Mascot Score and F) Sequest Score distribution for peptides that passed manual verification. Click here for file

Additional file 4: Figure S2 MS/MS spectra of 4 GSSPs mapping to intergenic regions that were used to find novel genes in *C. neoformans* var. *grubii.* Click here for file

Additional file 5: Table S3 List of peptides spanning exon junctions confirming the annotated splice junctions. Click here for file

Additional file 6: Table S4 Modifications in exon boundaries of genes using peptide evidence. Click here for file

Additional file 7: Figure S3 Refinement of annotated splice junction based on peptides mapping to introns and exon – intron junctions A) GSSPs mapped to intronic (YVEFNLVYDR) and exon – intron junction (FGLNTPGAR) of CNAG_02460. Based on comparative genomics and predicted models, annotated exon boundaries of CNAG_02460 were revised. B) Representative MS/MS spectrum of intronic peptide is provided. Click here for file

Additional file 8: Table S5 Modifications in gene boundaries using peptide evidence. Click here for file

Additional file 9: Figure S4 Extension of gene model using intergenic peptide A) Peptide mapped to upstream of gene CNAG_05600. Extension of protein comprising this peptide is supported by predicted models and homologous proteins in *C. neoformans* var. *neoformans*. B) Representative MS/MS spectrum of the intergenic peptide is illustrated. Click here for file

Additional file 10: Table S6 List of peptides providing protein-coding evidence of UTRs of genes. Click here for file

Additional file 11: Figure S5 Protein-coding evidence for regions annotated as UTR A) Three peptides mapped to 3′ UTR of CNAG_00768. Orthologous proteins from Cryptococcus spp. and prediction models support the extension of protein at C – terminus. B) Representative MS/MS spectrum of EGYYELCIK peptide is provided. Click here for file

Additional file 12: Figure S6 MS/MS spectra of 4 GSSPs mapping to exonic regions of annotated gene that were used to correct the translational frame of the genes. Click here for file

Additional file 13: Table S7 List of GSSPs mapping to exonic region of genes aiding in correction of translational frame. Click here for file

Additional file 14: Table S8 List of primers used for RT-PCR based validation of novel and revised gene models. Click here for file
