# Supplementary material for: Proteogenomic analysis of pathogenic yeast Cryptococcus neoformans using high resolution mass spectrometry
Source: Clin Proteomics. 2014 Feb 3;11(1):5. doi: 10.1186/1559-0275-11-5 (PMC3915034; doi:10.1186/1559-0275-11-5)
Supplement: Additional file 14: Table S8 — List of primers used for RT-PCR based validation of novel and revised gene models. [file 1559-0275-11-5-S14.docx]

**Additional file 14: Table S8: List of primers used for RT-PCR based validation of novel and revised gene models**

| **Transcript Identifier** | **Forward Primer (5’ – 3’)** | **Reverse Primer (5’ – 3’)** |
| --- | --- | --- |
| CNAG_IOB_PROT1 | CAGACAAGCAGACTCTATCCGAAC | CCCAATCCAGACCCTACAATC |
| CNAG_IOB_PROT2 | CCTCCTCATCAAGTCACTCTCC | AGCAGACCTAATTTCAGCCATC |
| CNAG_IOB_PROT3 | CTGTTCGATAATGACGCTGATG | GTCATCGTTCCTTTCCTGTTG |
| CNAG_IOB_PROT4 | ACTCACCTTCCAGTTCATCACTTC | TGCGTCCAAATCTCTTCCTC |
| CNAG_00409_GE1 | TTGAACGGAGAAAGGAGAGAAG | TACGCCTTGCGTCTACAAATGA |
| CNAG_01324_GE2 | AGGACTCAAAGAAGGAAGAAGACC | TAGATGATACGCTGGGCAAAGA |
| CNAG_01613_GE3 | GTTGAGGGCAAGAATAGTGATGAG | GTTCCTCTTCTGACGGGATTATGT |
| CNAG_01660_GE4 | GTCTCGCTTCCGAACTCACC | TGAGGAGCAGTAATAGCACAGAAC |
| CNAG_01895_GE5 | CGTTGTACCTCACCGAAGACC | CACATTCTGCTCAGTGTTCTCTACC |
| CNAG_02001_GE6 | CCTCTTTCGGCACACACAC | CGAAGCTCTGCGACAAAC |
| CNAG_05600_GE7 | CAGGACTCCGTCTAGGTTGG | CAGTGCTGCCTCTACCTCTT |
| CNAG_07936_GE8 | TTCGCTGCTGTATCAGGTAATG | GTTGCTCTCCTGCCACAATC |
| CNAG_02122_NE1 | GCAAAGTGTGCAAAGAGGTATG | CTCGGCGTACCATTAAGAGAAG |
| CNAG_02773_NE2 | AGGGTGTAATAGACGGTTTGCTTG | GAGTCCATCTCTTCTTCTGAGGTT |
| CNAG_03648_NE3 | AGCTCAGACAGAGAAGCAACGA | CATAGCCGTAGACCCTTCCA |
| CNAG_03920_NE4 | CCTCTCTTCTCCGATCAACTAGAC | GATGGTAGCGCACTTGACAC |
| CNAG_06526_NE5 | ATACCTGAATCGAGCCGAAC | ATCAGATCGGGTGGAAATACTC |
| CNAG_06533_NE6 | GGATTGTTCTTGTTCATCCTCTCC | TATCCAAGCCAAACTTCCTCTC |
| CNAG_02396_NE7 | CTCAATCTGGCCTGCATCTC | CATACCTTCTTTCTCGATCCCTGT |
| CNAG_02237_EE1 | GCCGACTACGACGACTATCC | GGTTCGGAGACCATTCCA |
| CNAG_02440_EE2 | AAGGAAAGGAAGACATCCCAAG | GTACATAATGGTGCGGAATGAG |
| CNAG_02460_EE3 | GTCTACAAGCACATGAACGACA | CGGCAACGACATCAAGATAC |
| CNAG_04735_EE4 | CGAGTTCCTCTTCGTCTTGTCTC | GTCGCTCCTAACACCACCAC |
| CNAG_05379_EE5 | GGTACTCTGGGCGAAGGTTG | CAGAAGAAGGCTCGTAGGTGTAG |
| CNAG_07372_EE6 | GCTACAAGGTCGTCCAGAAAC | ACAAATCATCCTGACACCTCTCC |
| CNAG_07524_EE7 | TCCTTACAGCCCATTCTATCTCTC | GCAAATACCACCTTTGACCTAACC |
| CNAG_01107_EE9 | GCGAAGAAATGAAGCGAAAG | GTAGTTCTCGCAGACCACAGGA |
| CNAG_01298_EE10 | CAGAAACTGTTCACGCCATTC | AACTCGTCCGTTCATCTCATCT |
| CNAG_01579_EE11 | CGGTATCCTAGACCATAACCTTACC | AGGTTCACTCGTTGCCTTAATC |
| CNAG_01601_EE12 | GTGGACCGACTGCAAAGAATG | AGGAGAGACCGATCAGAGATGAGA |
| CNAG_01813_EE13 | GGTCACGAAGCCCAACTCTAC | ACATCCATCCAGAACCCTTG |
| CNAG_00663_EE14 | AAGACGGTGCGGTGCTCTAC | CAATAACAACGCTCTTCCATCC |
| CNAG_01148_EE15 | AAGAAGGGTAAGAAGGCAGAGAAG | CTCGTCTTGCTGGATGTAGTTG |
| CNAG_01788_EE16 | ACCAAGTTTCGATGGACGAG | GTCCCTTGATACACTTTCTCCTTC |
| CNAG_02725_EE17 | ATGACAAAGCTCGCAGAAACC | AAGCACAAGATGAGCACCAAC |
| CNAG_03232_EE18 | AGTCTTCGTCGTGACCTTCC | CCCCGATCCAAATATACGAC |
| CNAG_03299_EE19 | CTTAGGTCGAAGCACGAGAATG | GTTGTTAGAGTCGCCAAGGAAG |
| CNAG_03678_EE20 | GACACTTTCACTCGCTTTCCTC | CCTTCTTCGCTATGTTCAATCC |
| CNAG_05975_EE21 | GTGGAGAAGGAACCCAAAGAG | ATAGGGATCGTAGGGAGGATAGA |
| CNAG_07719_EE23 | CGACCAGCCTTTGAATCCTC | TAATTGATGGCCGTTCCTCTAC |
| CNAG_07914_EE24 | AGACCAAGACCAACTTACTCGAAC | CTGACTTCTGCCTCGTTATCCT |
| CNAG_00936_EE25 | GATGAAGAAGGACAGGAGGATG | TCGGAGGTGTTGGATAGATAGG |
| CNAG_03735_EE27 | ACAACACTTTCCGAGAACGAAG | AGGTGACCAAAGTAGGAACGAG |
| CNAG_04149_EE28 | GTATAGAGTGTACGAGGGCTTTCTG | TCACGTCAGTTTCCTAACCTTG |
| CNAG_05792_EE29 | GTCGCACTTCTCTATGTTGGATG | AGTTTGGTAGAGTCCATCGTCTTG |
| CNAG_06123_EE30 | GTCGGTCCCAACCTCAAATAC | CCTCTCCGTAGTCGAGATACCA |
| CNAG_05920_EE31 | GCAGGTAGGAATTGGAAGAGTG | GTGGAGTTGTATTTCTGGAAGGAG |
